# Comprehensive pan‐genome analysis of *Lactiplantibacillus plantarum* complete genomes

**DOI:** 10.1111/jam.15199

**Published:** 2021-07-24

**Authors:** Francesco M. Carpi, Maria Magdalena Coman, Stefania Silvi, Matteo Picciolini, Maria Cristina Verdenelli, Valerio Napolioni

**Affiliations:** ^1^ Synbiotec S.r.l Camerino Italy; ^2^ School of Biosciences and Veterinary Medicine University of Camerino Camerino Italy; ^3^ Independent Researcher Gubbio Italy; ^4^ Genomic and Molecular Epidemiology (GAME) Lab School of Biosciences and Veterinary Medicine University of Camerino Camerino Italy

**Keywords:** evolutionary analysis, genomics, *Lactiplantibacillus plantarum*, microbiome, pan‐genome, probiotics

## Abstract

**Aims:**

The aim of this work was to refine the taxonomy and the functional characterization of publicly available *Lactiplantibacillus plantarum* complete genomes through a pan‐genome analysis. Particular attention was paid in depicting the probiotic potential of each strain.

**Methods and results:**

Complete genome sequence of 127 *L*. *plantarum* strains, without detected anomalies, was downloaded from NCBI. Roary analysis of *L*. *plantarum* pan‐genome identified 1436 core, 414 soft core, 1858 shell and 13,203 cloud genes, highlighting the ‘open’ nature of *L*. *plantarum* pan‐genome. Identification and characterization of plasmid content, mobile genetic elements, adaptative immune system and probiotic marker genes (PMGs) revealed unique features across all the *L*. *plantarum* strains included in the present study. Considering our updated list of PMGs, we determined that approximatively 70% of the PMGs belongs to the core/soft‐core genome.

**Conclusions:**

The comparative genomic analysis conducted in this study provide new insights into the genomic content and variability of *L*. *plantarum*.

**Significance and Impact of the Study:**

This study provides a comprehensive pan‐genome analysis of *L*. *plantarum*, including the largest number (*N* = 127) of complete *L*. *plantarum* genomes retrieved from publicly available repositories. Our effort aimed to determine a solid reference panel for the future characterization of newly sequenced *L*. *plantarum* strains useful as probiotic supplements.

## INTRODUCTION

Next‐generation sequencing (NGS) has extended our ability to understand complex biological phenomena, influencing drastically the experimental settings used up to the beginning of the second decade of the present century. Indeed, NGS allows the study of complex systems through the acquisition of an extensive amount of high‐quality data in relatively short time and low cost. At the same time, the implementation of solid and easy‐to‐use data retrieval systems from public databases is allowing the analysis of large data sets at almost zero cost. In this context, the ‐*omics* field, from genomics to proteomics and from transcriptomics to metagenomics, continues to expand unceasingly, improving both the pre‐existing analytical pipelines and our ability to interpret results deriving from very complex matrices. Nowadays, it is possible to contextualize the results coming from big data analyses with relative ease, thus providing findings with high translational impact.

In the present work, we aim to refine the taxonomy and the functional characterization of *Lactiplantibacillus plantarum* (*L*. *plantarum*), formerly known as *Lactobacillus plantarum*, a versatile Gram‐positive lactic acid bacterium, originally discovered in saliva, belonging to the large family of Lactobacillacae and present in a large range of environmental niches (Siezen et al., 2010). Notably, *L*. *plantarum*, one of the largest genomes known among the lactic acid bacteria, is able to survive gastric transit, thus easily colonizing the gut of human and other mammals (de Vries et al., 2006). Because of these properties, *L*. *plantarum* is considered one of the most used bacterial strains in food industry as probiotic and/or microbial starter. The utilization of *L*. *plantarum* strains, characterized by their long history in food fermentation, is an emerging field in the designing of value‐added foods (Behera et al., 2018). Indeed, *L*. *plantarum* strains have been used to produce new functional (traditional/novel) foods and beverages with improved nutritional and technological features (Behera et al., 2018). *Lactiplantibacillus plantarum* strains were identified from many traditional foods and characterized for their systematics and molecular taxonomy, enzyme systems (α‐amylase, esterase, lipase, α‐glucosidase, β‐glucosidase, enolase, phosphoketolase, lactase dehydrogenase, etc.), and bioactive compounds (bacteriocin, dipeptides, and other preservative compounds) (Behera et al., 2018). Moreover, recent studies on microbiome composition, both in humans and in animal models, showed that *L*. *plantarum* strains possess clinically beneficial properties, to ameliorate dysbiosis states occurring in several medically relevant conditions, such as obesity (Soundharrajan et al., 2020) or cognitive dysfunction in major depression (Rudzki et al., 2019). A recent work from our group has also demonstrated that *L*. *plantarum* is able to prevent colonization of the urogenital tract by relevant pathogens such as *Candida* strains (Coman et al., 2015).

In this context, given its high translational potential in food industry and in clinical settings as well, a comprehensive analysis of deposited *L*. *plantarum* strain genomes, both at phylogenetical and functional level, by means of pan‐genome analysis, may provide useful insights into the different properties of *L*. *plantarum* strains. We expect this will also allow for a better selection of *L*. *plantarum* strains to be used in industrial settings and for an improved understanding of their effects on human health *tout court*. Moreover, a comprehensive pan‐genome analysis of *L*. *plantarum* complete genomes may be serving as a reference, to help characterizing and identifying the beneficial properties of new isolated strains, potentially introducible in probiotic‐supplementation formulas or in the production of functional foods.

Notably, two recent studies have already performed a pan‐genome analysis of *L*. *plantarum*, considering the genome of 108 and 49 strains, respectively (Choi et al., 2018; Evanovich et al., 2019). In addition, another recent study reported the comparative pan‐genome analysis of five different *Lactobacillus* species (i.e. *L*. *reuteri*, *L*. *delbrueckii*, *L*. *plantarum*, *L*. *rhamnosus* and *L*. *helveticus*), including 124 genomes of *L*. *plantarum* (Inglin et al., 2018). However, the main limitation of those studies lies in the fact that most of the genomes included in their analyses were not complete, providing the analysed genomes just at their ‘draft’ stage. The use of poorly assembled genomes, such as the ones provided in their draft stages, can intrinsically lead to analytical biases, therefore to incorrect taxonomic and/or functional characterization of the different strains.

Herein, we provide a comprehensive pan‐genome analysis of complete *L*. *plantarum* genomes (*N* = 130), comparing the most updated genomic information with the previous findings, that mostly leveraged publicly available *L*. *plantarum* draft genomes, and QCing them according to the most updated pan‐genome analysis pipelines (Wu et al., 2021). This will greatly expand our knowledge of *L*. *plantarum* biology, while providing, at the same time, a direct validation of the previous published findings.

## MATERIALS AND METHODS

### Lactiplantibacillus plantarum complete genome sequence retrieval

The complete genome sequence of *L*. *plantarum* strains was downloaded from the National Center for Biotechnology Information (NCBI), under the ‘Assembly’ section, querying for ‘*Lactiplantibacillus plantarum*’. Of the 541 available assemblies (July 2020), 130 were reported to have a complete assembly, thus with guaranteed full genome representation, which were used in the present work. Details regarding the identification, isolation source and sequencing of the samples are described in Table S1. Among these 130 genomes, we removed three *L*. *plantarum* strains (CNEI‐KCA5, KLDS1.0391 and SN13T) that were missing the RefSeq assembly because of detected anomalies, as reported by NCBI (e.g. missing tRNA genes, many frameshifted proteins). Thus, we finally obtained 127 strains of *L*. *plantarum* for subsequent analyses.

### Genome annotation

The 127 complete genomes of *L*. *plantarum* were annotated using the Prokaryotic Genome Annotation System (Prokka), v1.14.5 (Seemann, 2014 **)** and further refined using eggNOG‐mapper v2 (Huerta‐Cepas et al., 2017) using precomputed eggNOG v5.0 clusters and phylogenies (Huerta‐Cepas et al., 2019). Functional annotation was performed through the Rapid Annotations Subsystems Technology (RAST) (Aziz et al., 2008).

### Phylogenetic and average nucleotide identity analysis

According to Wu et al., (2021), the inclusion of confounding strains may introduce important biases that will greatly influence the interpretation of the pan‐genome analyses. Thus, as suggested by Wu et al., (2021), we first determined the phylogenetic relationship among the 127 *L*. *plantarum* strains using OrthoFinder v2.4.0 (Emms & Kelly, 2019) with default parameters, using the protein sequences obtained from Prokka annotation. Then, we performed average nucleotide identity (ANI) analysis using FastANI v1.31 (Jain et al., 2018) with default parameters, using the nucleotide sequences directly retrieved from NCBI.

### Pan‐genome analysis

After determining the presence of potential confounding strains from the phylogenetic and ANI analysis of the 127 *L*. *plantarum* strains considered, we used Roary v3.11.2 (Page et al., 2015) to perform pan‐genome analyses using the GFF3 files generated by Prokka (Seemann, 2014). Accordingly, we obtained four different classes of genes belonging to ‘core’ (99% ≤strains ≤100%), ‘soft core’ (95%≤ strains <99%), ‘shell’ (15%≤ strains <95%) and ‘cloud’ (0%≤ strains <15%) groups, respectively. Thus, we aligned the core genomes of *L*. *plantarum* strains using Parsnp v1.5.3 (Treangen et al., 2014), calling the single‐nucleotide polymorphisms (SNPs) and determining the core genome phylogeny.

### Identification of clinically relevant genomic elements and functional clustering

The information regarding the presence of plasmids was retrieved from NCBI Assembly page for each strain. To further explore the landscape of non‐chromosomal genome sequences, we also queried PlasmidFinder v.2.0.1 for the presence of previously annotated replicons, considering the ‘Enterobacteriaceae +Gram‐positive’ database, setting an 80% threshold for minimum percentage of identity and a 60% threshold for minimum coverage (Carattoli et al., 2014). PHAge Search Tool Enhanced Release (PHASTER) (Arndt et al., 2016) was used to screen for prophage specifying DNA regions within the genome of all strains. The bacterial genome sequence in FASTA format was used as input to detect the genes responsible for bacteriocin production using BAGEL4 software (van Heel et al., 2018). Clustered Regularly Interspaced Short Palindromic Repeats (CRISPR) and their associated (Cas) protein were data mined using CRISPRCasFinder (Couvin et al., 2018).

Using the Comprehensive Antibiotic Resistance Database 2020 (Alcock et al., 2020), we further determined the presence of acquired antibiotic resistance genes, prophages, bacteriocins and plasmids in the *L*. *plantarum* strains analysed.

### Statistical analysis

Fisher's exact test was applied to contingency tables using R, with a statistical significance threshold set at *p* < 0.05 (two sides).

## RESULTS

### Main genomic features of *L. plantarum* pan‐genome

**FIGURE 1:**
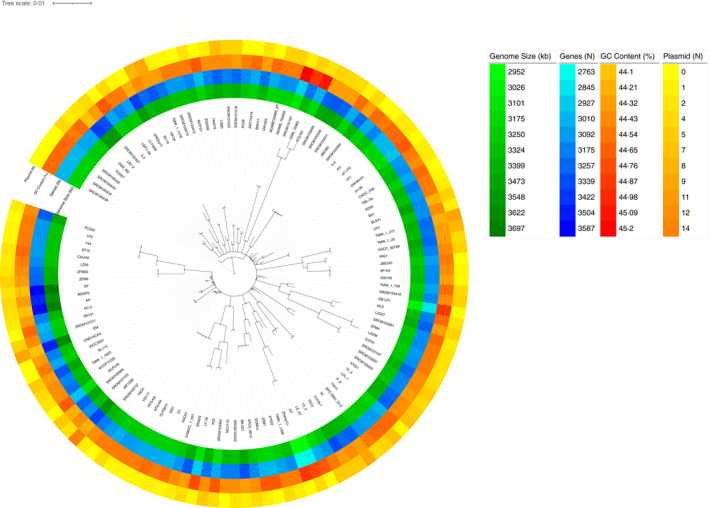
Phylogenetic tree obtained by OrthoFinder and genomic features of the 127 *Lactiplantibacillus plantarum* genomes. The Circos heatmap from the inside to the outside report the genome size, gene number, GC content and number of plasmids for each strain

**FIGURE 2:**
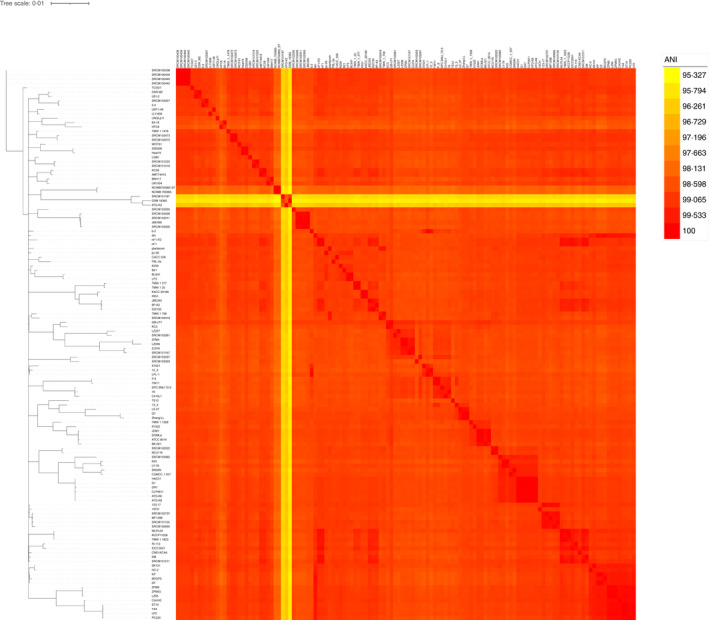
Correlation matrix of average nucleotide identity for the 127 *Lactiplantibacillus plantarum* genomes obtained by FastANI

Compared with previous studies reporting pan‐genome analysis of *L*. *plantarum* strains (Choi et al., 2018; Evanovich et al., 2019; Inglin et al., 2018), our study includes 107, 80 and 110 additional strains, respectively, for which the complete genome is now publicly available (Table S1). The analysis of 127 *L*. *plantarum* genomes by Orthofinder (Figure 1) and FastANI (Figure 2) did not show the presence of mis‐assigned strains to the species. Thus, the full data set was considered in the subsequential analyses.

Nonetheless, both the analytic tools, when providing the relative phylogenetic trees, clearly displayed the presence of four (SRCM100438, SRCM100434, SRCM100440 and SRCM100442 by Orthofinder, Figure 1) and three strains (SRCM101187, ATG‐K2 and DSM 16365 by FastANI, Figure 2), respectively, that clustered separately from the rest of whole data set. The genome of the four phylogenetically distant strains identified by Orthofinder were all provided by the Microbial Institute for Fermentation Industry located in South Korea and apparently did not show any particular genomic feature to be considered outliers or mis‐assigned strains to the *L*. *plantarum* species. Conversely, the three phylogenetically distant strains detected by FastANI represent the ones with the highest GC content among all the *L*. *plantarum* strains analysed.

The average full genome size and GC content of the 127 *L*. *plantarum* strains were 3.32Mb and 44.5%, respectively, with a number of plasmids ranging from 0 to 14 (Figure 1). The average non‐chromosomal genome size was 119Kb, where 17 out of the 127 considered strains (21.3%) were plasmid‐free.

### *Lactiplantibacillus plantarum pan*‐*genome analysis*

**FIGURE 3:**
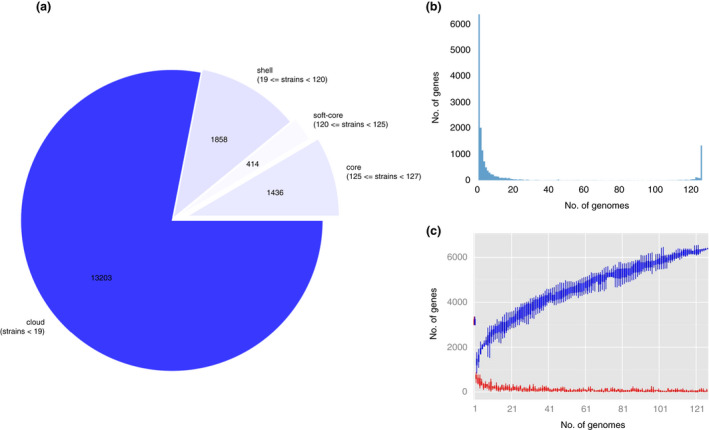
*Lactiplantibacillus plantarum* pan‐genome. (a) The number of genes belonging to the core, the soft core, the shell or the cloud of the *L*. *plantarum* species is pictured as a pie chart; (b) gene frequency versus genome number; (c) representation of *L*. *plantarum* gene content (extrapolated median‐based line) according to how the pan‐genome varies as genomes are added in random order to the analysis. The blue line represents unique genes; the red line represents new genes

Roary analysis of *L*. *plantarum* pan‐genome identified 1436 core, 414 soft core, 1858 shell and 13,203 cloud genes, respectively, out of 16,911 total genes (Figure 3a). The large number of cloud genes implies that a large heterogeneity exists among the 127 *L*. *plantarum* strains considered, highlighting the ‘open’ nature of *L*. *plantarum* pan‐genome (Figure 3b). Nonetheless, we noticed that the number of new genes is progressively decreasing, proportionally to the number of genomes included in the analysis, while approximatively 30 new genes are continuously added for each additional genome after the first 100 genomes considered (Figure 3c).

**FIGURE 4:**
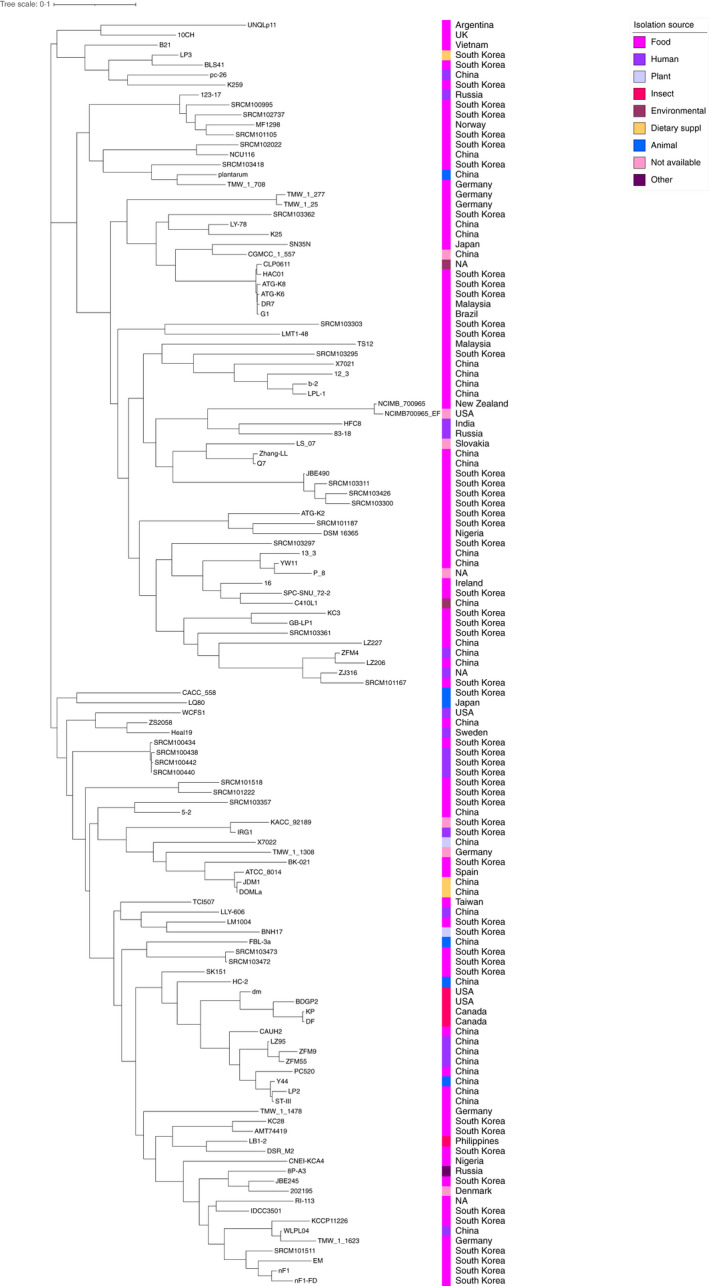
Phylogenetic tree of the 127 *Lactiplantibacillus plantarum* strains. Tree based on orthologous genes found by Roary among the strains. The length of each branch is proportional to the number of orthologs found. Isolation source and geographical provenience of each strain are reported

**FIGURE 5:**
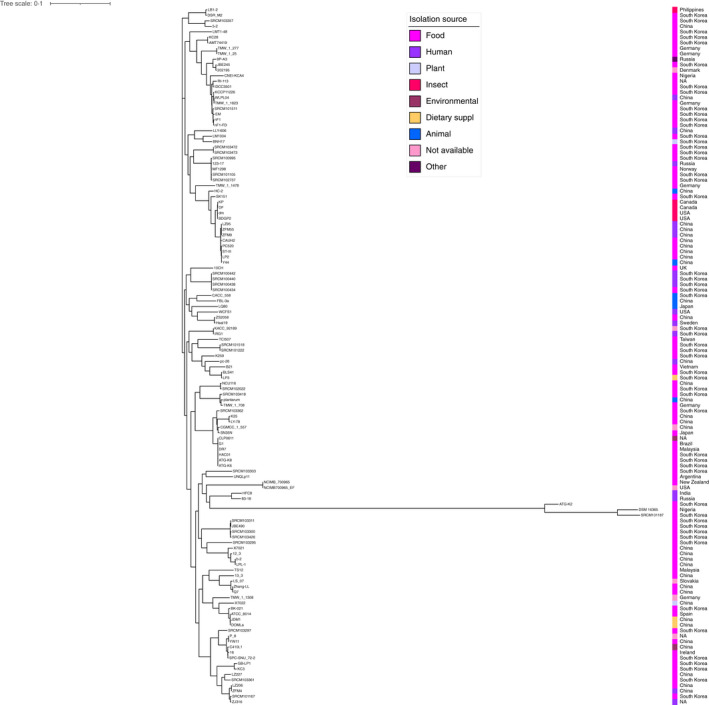
Phylogenetic tree of the 127 *Lactiplantibacillus plantarum* strains. Tree based on single‐nucleotide polymorphisms (SNPs) identified by Parsnp among the strains. The length of each branch is proportional to the number of SNPs found. Isolation source and geographical provenience of each strain are reported

The phylogenetic tree based on orthologous genes found by Roary was compared with the one obtained from the core‐genome analysis performed using Parsnp v1.5.3 (Treangen et al., 2014) (Figures 4 and 5). Both phylogenetic trees defined three main clades that showed a different strain distribution, both at qualitative and quantitative level (Table 1). Indeed, none of the strains belonging to the first clade were consistent between the two phylogenetic trees; in addition, strain distribution across the three clades was significantly different (*p* = 0.036). Strain distribution across the three clades differed significantly only for the ones determined by the phylogenetic tree based on orthologous genes, when stratified according to isolation source category (*p* < 0.018, Table 1).

**TABLE 1:** *Lactiplantibacillus plantarum* strain distribution according to the three main phylogenetic clades identified using orthologous genes and core genome single‐nucleotide polymorphisms (SNPs)

		Isolation source category								Total
Phylogenesis	Clade	Animal	Dietary suppl.	Environmental	Food	Human	Insect	Plant	NA +other	
A										
Orthologous genes^a^	1	—	1 (14.3%)	—	5 (71.4%)	1 (14.3%)	—	—	—	7
	2	1 (1.7%)	—	2 (3.3%)	48 (80.0%)	5 (8.3%)	—	—	4 (6.7%)	60
	3	5 (8.3%)	2 (3.3%)	—	31 (51.7%)	11 (18.3%)	5 (8.3%)	2 (3.3%)	4 (6.7%)	60
Core SNPs^b^	1	—	—	—	3 (75.0%)	—	1 (25.0%)	—	—	4
	2	2 (4.7%)	—	—	28 (65.1%)	6 (14.0%)	4 (9.3%)	1 (2.3%)	2 (4.7%)	43
	3	4 (5.0%)	3 (3.8%)	2 (2.5%)	53 (66.3%)	11 (13.8%)	—	1 (1.3%)	6 (7.5%)	80
Total		6 (4.7%)	3 (2.7%)	2 (1.6%)	84 (66.1%)	17 (13.4%)	5 (3.9%)	2 (1.6%)	8 (6.3%)	127

		Geographical area								Total
Phylogenesis	Clade	Africa	East Asia	Europe	North America	Oceania	South America	South Asia	NA	
B										
Orthologous genes^c^	1	—	5 (71.4%)	1 (14.3%)	—	—	1 (14.3%)	—	—	7
	2	1 (1.7%)	42 (70.0%)	8 (13.3%)	1 (1.7%)	1 (1.7%)	1 (1.7%)	3 (5.0%)	3 (5.0%)	60
	3	1 (1.7%)	46 (76.7%)	7 (11.7%)	5 (8.3%)	—	—	—	1 (1.7%)	60
Core SNPs^d^	1	—	4 (100.0%)	—	—	—	—	—	—	4
	2	1 (2.3%)	29 (67.4%)	8 (18.6%)	4 (9.3%)	—	—	—	1 (2.3%)	43
	3	1 (1.3%)	60 (75.0%)	8 (10.0%)	2 (2.5%)	1 (1.3%)	2 (2.5%)	3 (3.8%)	3 (3.8%)	80
Total		2 (1.6%)	93 (73.2%)	16 (12.6%)	6 (4.7%)	1 (0.8%)	2 (1.6%)	3 (2.7%)	4 (3.1%)	127

(A) *Lactiplantibacillus plantarum* strain distribution by isolation source category; (B) *Lactiplantibacillus plantarum* strain distribution by geographical area. Differences in strain distribution were tested by Fisher's exact test: ^a^ *p*‐value =0.018; ^b^ *p*‐value =0.258; ^c^ *p*‐value =0.296; ^d^ *p*‐value = 0.649.

Notably, the SNP‐based, core‐genome phylogenetic tree confirmed the peculiarity of SRCM101187, ATG‐K2 and DSM 16365 strains, already highlighted by FastANI as potential outliers (Figure 2).

### *Mobile genetic elements and adaptative immune systems in Lactiplantibacillus plantarum pan*‐*genome*

Determining and characterizing mobile genetic elements (MGEs) are of paramount relevance when defining the probiotic potential for a given strain because they largely contribute to antibiotic resistance (Tait, 1993) and to horizontal gene transfer (Rankin et al., 2011).

MGEs include plasmids, transposons and bacteriophages. Conversely, CRISPR‐Cas elements and bacteriocins represent adaptative immune systems to protect against deadly consequences from MGEs or competing bacteria (Cotter et al., 2005; Klaenhammer, 1993; Peters et al., 2017).

PlasmidFinder v.2.0.1, which leverage a comprehensive, curated database of plasmid replicons, allowed the identification of plasmids already annotated (Carattoli et al., 2014). Out of 127 strains, we were able to annotate 127 plasmid‐replicons belonging to nine different classes (rep38_CP002655; rep38_CP005943; repUS73_CP002654; rep28_CP003162; rep28_CP005948; repUS73_CP002654; repUS64_JN601038; rep32_AL592102; repUS75_CP002393) distributed across 67 *L*. *plantarum* strains (Table S2). The most represented plasmid‐replicon was rep38_CP005943, found in 24 strains (total copies =32), which was originally annotated as *L*. *plantarum* P‐8 plasmid LBPp1.

Bacteriophage identification by PHASTER (Arndt et al., 2016) showed that the sequences of bacteriophage origin varied from 35Kb (strain SRCM101511) to 300 kb (strain DF), that is, about 1–8% of the size of the *L*. *plantarum* genomes. Bacteriophage proteins (DNA packaging protein, holin protein, lysin, tail, capsid, protease, terminase and integrase) and hypothetical proteins were the most frequent ones. The bacteriophages most encountered were Sha1 and Phig1, both isolated from *L*. *plantarum*. It suggests a high gene transfer rate between the strains. Table S3 shows in detail all the results from PHASTER (Arndt et al., 2016).

**FIGURE 6:**
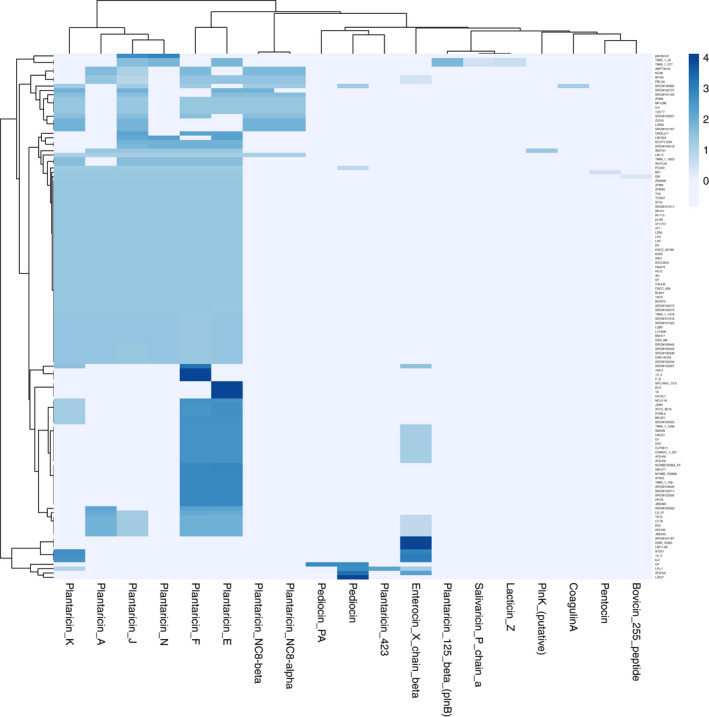
Bacteriocin identification/annotation heatmap of the 127 *Lactiplantibacillus plantarum* genomes. The heatmap reports the normalized scalar values of bacteriocin identification/annotation by BAGEL4 for each strain, using as lowest threshold a sequence homology of 50%

The results of bacteriocin identification/annotation are shown in Figure 6. All the strains harboured at least one bacteriocin gene, especially of Plantaricin ‐A, ‐K, ‐J, ‐N, ‐E and ‐F classes. Notably, only *L*. *plantarum* Q7 strain has Pediocin PA‐1, the most extensively studied class Ila (or pediocin family) bacteriocin, which shows a particularly strong activity against *Listeria monocytogenes*, a foodborne pathogen of special concern among the food industries (Rodríguez et al., 2002).

A total of 101 *L*. *plantarum* strains carried at least one CRISPRs array or one Cas cluster (Table S4). The number of CRISPR arrays varied from five to one, with ATG‐K6 and DSM_16365 strains carrying the highest number of CRISPRs arrays each (*N* = 5), along with two different Cas cluster types, CAS‐TypeIIA and CAS‐TypeIE, respectively. Conversely, two strains, SRCM103472 and TMW 1.1478, displayed no CRISPRs arrays, while harbouring a CAS‐TypeIIA and a CAS clusters. Overall, the majority (*N* = 61) of the identified 101 *L*. *plantarum* strains carrying CRISPR‐Cas systems components, harboured a single, small CRISPRs array (with 3 or less spacers). Similarly, only 41 strains included a single Cas cluster in their genome, except for LQ80 strain, which harboured two Cas clusters. Of the 41 strains with identified Cas clusters, 35 had CAS‐TypeIIA, whereas the remaining ones had CAS‐TypeIE (*N* = 2), CAS (*N* = 2) and CAS‐TypeIA.

Only the *L*. *plantarum* 12_3 strain was identified as carrier of acquired antibiotic resistance genes, with the presence of an ANT(6) gene, producing an aminoglycoside antibiotic.

### *‘Probiotic marker genes’ in L. plantarum pan*‐*genome*

A probiotic bacterium should have the ability to survive, and transiently persist, in the gastrointestinal tract where has to be able to exert a beneficial effect. Apart from MGEs and adaptative immune systems genes, the ability to resist host stressful conditions and to hydrolyse bile salts is of paramount relevance when determining the key genes defining a candidate bacterium with potential probiotic potential.

Lebeer et al., (2008) provided a comprehensive summary of *Lactobacillus* genes involved in stress resistance, active metabolism in the host, adhesion and putative probiotic functions. Combining their list with the results obtained from more recent papers, we created an updated list of ‘probiotic marker genes’ (PMGs) responsible for stress resistance (acid, osmotic, oxidative, temperature), bile salt hydrolase activity, adhesion ability and gut persistence to find out any unique features across all the *L*. *plantarum* strains included in the present study. The annotation and the presence/absence status of the 75 identified PMGs are reported as Table S5.

According to the comparative pan‐genome analysis carried out using Roary, we determined that approximatively 70% of the considered PMGs belongs to the core/soft‐core genome (95%≤ strains ≤100%). Thus, we focused on the ‘shell’ and ‘cloud’ PMGs because they may highlight strain‐specific peculiarities in their probiotic potential. In particular, we noticed that five PMGs (*bshA*, *oppA_4*, *srtA*, *xylA*, *gla_2*) were present, individually, in less than five strains (Table 2). As example, the *bshA* gene, responsible for bile tolerance (Lambert et al., 2008; Lebeer et al., 2008), was present only in the *L*. *plantarum* 16 strain. Dispensable gene involved in gut persistence (*xylA*) was observed exclusively in three strains (SRCM103472, SRCM103473, TMW_1_1478) (Table 2). Similarly, PMG involved in adhesion ability (*srtA*) (Turpin et al., 2012) was found in two strains, DF and KP (Table 2).

**TABLE 2:** Probiotic marker genes (PMGs) in *Lactiplantibacillus plantarum* shell and cloud genome. PMGs were classified according to their presence or absence from the *L*. *plantarum* shell/cloud genome

Category	Gene	Annotation	Strain	*N*
*Presence*				
Acid stress	*clpP_1*	ATP‐dependent Clp protease proteolytic subunit	12_3; ATCC_8014; B21; BK‐021; BLS41; CGMCC_1_557; DOMLa; HC‐2; Heal19; JDM1; KC3; LMT1‐48; LPL‐1; LZ206; LZ227; NCU116; RI‐113; SN35N; SRCM100434; SRCM101167; SRCM102737; SRCM103295; SRCM103303; SRCM103362; SRCM103418; TCI507; TMW_1_1308; TMW_1_1478; TMW_1_708; UNQLp11; ZFM4; ZJ316; b‐2	33/127
Bile resistance	*bshA*	Bile salt hydrolase	16	1/127
Bile resistance	*oppA_4*	Oligopeptide‐binding protein OppA	SRCM101518	1/127
Bile resistance/adhesion	*srtA*	Sortase A	DF; KP	2/127
Gut persistence	*xylA*	Xylose isomerase	SRCM103472; SRCM103473; TMW_1_1478	3/127
Osmotic stress	*gbuB*	Glycine betaine/carnitine transport permease protein GbuB	16; BLS41; CACC_558; CGMCC_1_557; DSR_M2; K259; KC3; LP3; LZ227; LZ95; MF1298; NCIMB700965_EF; NCIMB_700965; NCU116; PC520; RI‐113; SN35N; SRCM101105; SRCM101167; SRCM101511; SRCM103357; SRCM103361; ST‐III; TMW_1_1623; TMW_1_25; TMW_1_277; X7021; X7022; Y44; ZFM4; ZFM55; ZFM9; ZJ316	33/127
Osmotic stress	*gla_2*	Glycerol facilitator‐aquaporin gla	ATG‐K2; Heal19; LZ227; PC520	4/127
*Absence*				
Bile resistance	*glf*	UDP‐galactopyranose mutase	12_3; 13_3; 16; 5‐2; 83‐18; ATCC_8014; b‐2; C410L1; CNEI‐KCA4; DOMLa; GB‐LP1; HFC8; JDM1; K25; LQ80; LY‐78; NCIMB700965_EF; NCIMB_700965; P_8; plantarum; Q7; RI‐113; SPC‐SNU_72‐2; SRCM101187; SRCM101222; SRCM103297; SRCM103303; SRCM103362; SRCM103418; TMW_1_1308; TMW_1_25; TMW_1_277; TMW_1_708; WLPL04; X7022; YW11; Zhang‐LL	37/127
Bile resistance	*cbh/bsh*	Choloylglycine hydrolase/ Bile salt hydrolase	12_3; GB‐LP1; HFC8; KC3; NCIMB700965_EF; NCIMB_700965; Q7; Zhang‐LL	8/127
Bile resistance	*oppA_3*	Oligopeptide‐binding protein OppA	83‐18; ATG‐K6; ATG‐K8; CGMCC_1_557; CLP0611; DR7; G1; HAC01; HFC8; K25; K259; LM1004; LS_07; LY‐78; NCIMB700965_EF; NCIMB_700965; NCU116; Q7; SN35N; SRCM101167; SRCM101187; SRCM102022; SRCM103295; SRCM103303; SRCM103362; TCI507; TMW_1_1478; TMW_1_25; TMW_1_277; ZJ316; Zhang‐LL	31/127
Bile resistance	*dps*	DNA protection during starvation protein	83‐18; BLS41; CGMCC_1_557; HFC8; LZ227; NCIMB700965_EF; NCIMB_700965; SN35N; SRCM103303; SRCM103362; TMW_1_25; TMW_1_277; X7022;	13/127
Osmotic stress	*glpF_1*	Glycerol uptake facilitator protein	12_3; ATG‐K2; LMT1‐48; LZ227; SRCM101187; SRCM103295; SRCM103297; SRCM103303; X7021	9/127

## DISCUSSION

This study provides a comprehensive pan‐genome analysis of *L*. *plantarum*, including the largest number (*N* = 127) of complete *L*. *plantarum* genomes retrieved from publicly available repositories. Our effort aimed to determine a solid reference panel for the future characterization of newly sequenced *L*. *plantarum* strains useful as probiotic supplements. Indeed, we paid particular attention in depicting the probiotic potential of each strain included in the analysis, through the identification and characterization of their plasmid content, MGEs, adaptative immune system and PMGs. Moreover, the dissection of *L*. *plantarum* pan‐genome into the four different gene categories (‘core’, ‘soft core’, ‘shell’ and ‘cloud’) will facilitate genetic engineering strategies for genomic reduction/optimization. Furthermore, our results showed that phylogenetic tree analyses represent a powerful methodology to predict potential outliers and to elucidate the real isolation source of the strains by helping to address mis‐annotation and cross‐contamination issues.

Understanding the origin of isolation of each strain and their niche‐specific adaptation can be of particular relevance for their further applications to improve probiotic efficacy and industrial workhorses.

Several important features separate our work from previous studies looking at the pan‐genome or for general comparative genomic analysis of *L*. *plantarum* (Choi et al., 2018; Evanovich et al., 2019; Inglin et al., 2018).

First, and most critically, we considered only the *L*. *plantarum* strains for which a complete genome was available. Indeed, it becomes obvious that the inclusion of genomes at their draft stage in a pan‐genome analysis can lead to severe biases that may compromise both data analysis and their interpretation. Not surprisingly, several recently developed tools are aiming to maximize bacterial pan‐genome analyses by adopting ad‐hoc strategies for the inclusion of draft genomes (e.g. Pan4Draft, PEPPAN; Veras et al., 2018; Zhou et al., 2020). Nonetheless, we deemed necessary to perform the first *L*. *plantarum* pan‐genome analysis using, exclusively, complete genomes to avoid any possible bias, while paving the way to a more exhaustive characterization of the genomic features of this bacterium.

Second, we focused on a pan‐genomic analysis centred on determining the probiotic potential of every *L*. *plantarum* strain considered. Previous works performed either a pan‐genome analysis of *L*. *plantarum* strains compared with other *Lactobacillus* species (e.g. *L*. *helveticus*, *L*. *delbrueckii*, *L*. *reuteri* and *L*. *rhamnosus*; Inglin et al., 2018) or a simple comparative genomic analysis within the *L*. *plantarum* strains available at the time of their respective studies (Choi et al., 2018; Evanovich et al., 2019). Thus, these works provided a quite fragmented picture on the genomic peculiarities relative to each strain considered in the analysis. Again, their results may be biased by the inclusion of draft genomes; as a matter of fact, our study includes 107, 80 and 110 additional strains compared with the aforementioned previous studies (Choi et al., 2018; Evanovich et al., 2019; Inglin et al., 2018); in particular, the number of overlapping genomes considered is 20, 47 and 17, respectively, highlighting both the novelty and higher reliability of our findings based on a much larger data set composed of uniquely complete genomes.

The comparative genomic analysis conducted in this study provide new insights into the genomic content and variability of *L*. *plantarum* confirming that the genomic screening of new strains is essential because the bacterial genomes are dynamic entities. Analyses of core, accessory and unique genes present in the genomes help in differentiating strains with different properties giving the opportunity to find potential probiotic candidates.

## CONFLICT OF INTEREST

No conflict of interest declared.

## AUTHOR CONTRIBUTIONS

Valerio Napolioni: conceptualization, methodology, genome collection, in silico analysis and writing the original draft. Francesco M. Carpi: in silico analysis, writing – original draft, review and editing. Magda M. Coman: visualization, review and editing. Stefania Silvi: visualization, review and editing. Matteo Picciolini: in silico analysis, review and editing. Maria Cristina: Verdenelli: conceptualization, review and editing.

## Supporting information

Table S1

Table S2

Table S3

Table S4

Table S5
